# Genetic polymorphism of Y-chromosome in Kazakh populations from Southern Kazakhstan

**DOI:** 10.1186/s12864-023-09753-z

**Published:** 2023-10-27

**Authors:** Yeldar Ashirbekov, Madina Seidualy, Arman Abaildayev, Albina Maxutova, Aigul Zhunussova, Ainur Akilzhanova, Kamalidin Sharipov, Zhaxylyk Sabitov, Maxat Zhabagin

**Affiliations:** 1 M. Aitkhozhin Institute of Molecular Biology and Biochemistry, Almaty, Kazakhstan; 2 National Center for Biotechnology, Astana, Kazakhstan; 3 Nazarbayev University, Astana, Kazakhstan; 4 Astana International University, Astana, Kazakhstan; 5 National Laboratory Astana, Astana, Kazakhstan; 6 Research Institute for Jochi Ulus Studies, Astana, Republic of Kazakhstan; 7 L.N. Gumilyov Eurasian National University, Astana, Republic of Kazakhstan

**Keywords:** Y-STR, Haplotype, Y-SNP, Haplogroup, Kazakh population, Southern Kazakhstan

## Abstract

**Background:**

The Kazakhs are one of the biggest Turkic-speaking ethnic groups, controlling vast swaths of land from the Altai to the Caspian Sea. In terms of area, Kazakhstan is ranked ninth in the world. Northern, Eastern, and Western Kazakhstan have already been studied in relation to genetic polymorphism 27 Y-STR. However, current information on the genetic polymorphism of the Y-chromosome of Southern Kazakhstan is limited only by 17 Y-STR and no geographical study of other regions has been studied at this variation.

**Results:**

The Kazakhstan Y-chromosome Haplotype Reference Database was expanded with 468 Kazakh males from the Zhambyl and Turkestan regions of South Kazakhstan by having their 27 Y-STR loci and 23 Y-SNP markers analyzed. Discrimination capacity (DC = 91.23%), haplotype match probability (HPM = 0.0029) and haplotype diversity (HD = 0.9992) are defined. Most of this Y-chromosome variability is attributed to haplogroups C2a1a1b1-F1756 (2.1%), C2a1a2-M48 (7.3%), C2a1a3-F1918 (33.3%) and C2b1a1a1a-M407 (6%). Median-joining network analysis was applied to understand the relationship between the haplotypes of the three regions. In three genetic layer can be described the position of the populations of the Southern region of Kazakhstan—the geographic Kazakh populations of Kazakhstan, the Kazakh tribal groups, and the people of bordering Asia.

**Conclusion:**

The Kazakhstan Y-chromosome Haplotype Reference Database was formed for 27 Y-STR loci with a total sample of 1796 samples of Kazakhs from 16 regions of Kazakhstan. The variability of the Y-chromosome of the Kazakhs in a geographical context can be divided into four main clusters—south, north, east, west. At the same time, in the genetic space of tribal groups, the population of southern Kazakhs clusters with tribes from the same region, and genetic proximity is determined with the populations of the Hazaras of Afghanistan and the Mongols of China.

**Supplementary Information:**

The online version contains supplementary material available at 10.1186/s12864-023-09753-z.

## Background

**Fig. 1:**
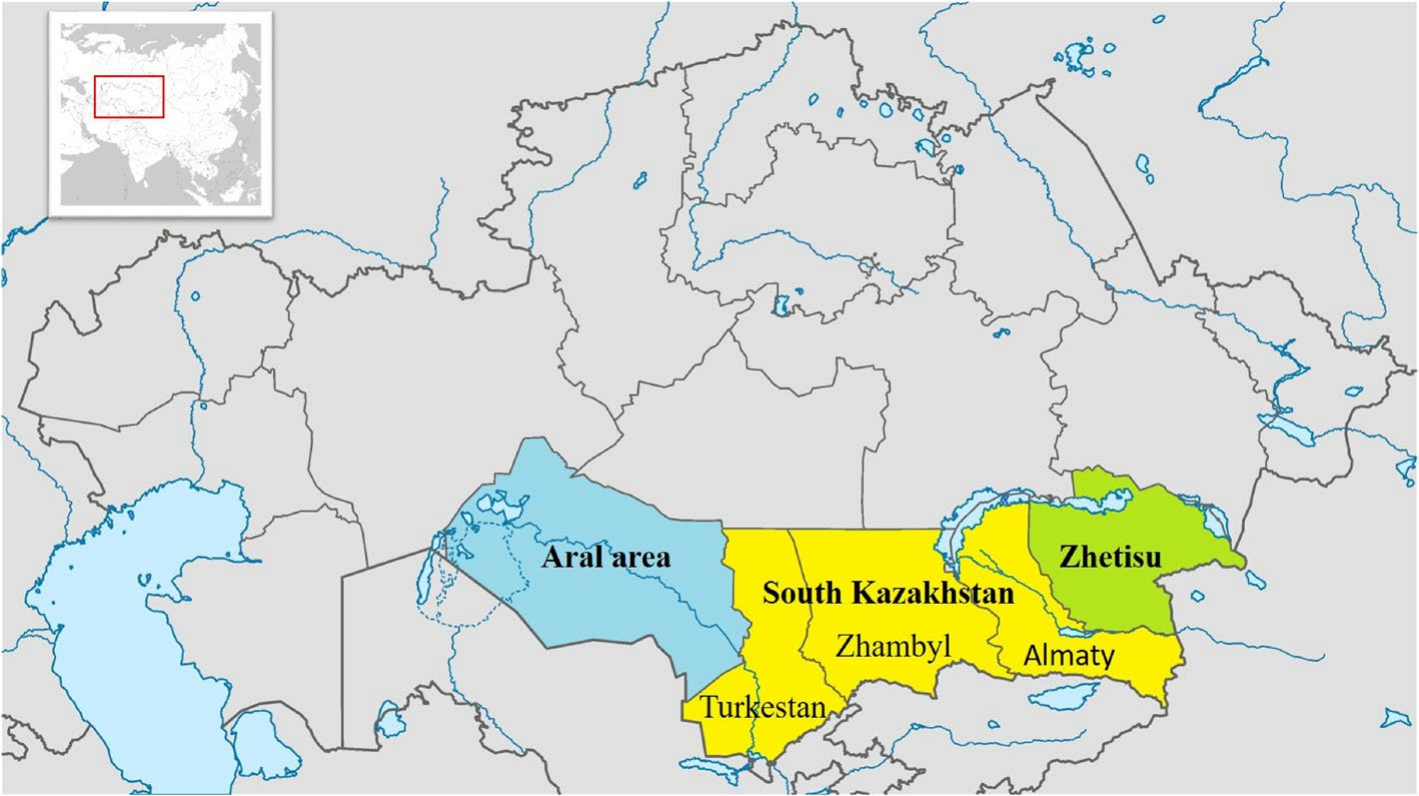
Historical and geographical sub-regions of the Southern area of Kazakhstan. Green represents Zhetisu, yellow represents the South (current areas of Almaty, Zhambyl, and Turkestan—the people investigated in this research), and Blue represents the Aral

Kazakhs are the world’s fourth biggest Turkic-speaking population (16 million people). The majority of them comprise the 13.5 million-person population of Kazakhstan in Central Asia [1]. The other is an indigenous national minority in China, Uzbekistan, Russia, and Mongolia. Almost one-third of Kazakhstan’s population lives in the country’s Southern area. The Southern region of the Kazakhstan stretches from the Betpak-Dala desert plateau and Lake Balkhash to the Dzungarian Alatau ranges in the north; from the Tien Shan western and northern foothills to the northern part of the Kyzylkum desert in the south; and from the Dzungarian Gates in the east to the Aral Sea in the west. The Southern region is subdivided into three historical and geographical regions: Zhetisu, the South, and the Aral. Three modern regions of Kazakhstan — Almaty, Zhambyl, and Turkestan — make up the South subdivision (Fig. 1).

The history of Kazakhstan’s southern area dates back to the early Paleolithic period. Ancient stone tools of the Klekton type were discovered in the thickness of the terrace on the right bank of the Arystandy River on the southern slopes of the Karatau Range [2].

Later, in the region, a unique Neolithic civilization was developed, the archaeological monument of which is the Karaungur cave. The material remains from this cave are characterized by the local South Kazakhstan Kelteminar civilization [3]. The traditions of the Andronovo cultural and historical community—the Tautara and Semirechye kinds—are pretty clearly traceable in the region throughout the Bronze Age [4]. There are several archaeological remains in the area from the time of the early nomads, who utilized iron extensively (VIII BC—V century AD). Among these are two Saki barrows of Zhetisu (V-III centuries BC)—Besshatyr and Issyk [5]; and an old Saka city Chirik-Rabat from the Aral Sea region [6]. During the third century BC to the fifth century AD, the region’s first state-type organizations are formed—Usun and Kangyui in the South and the Aral Sea sub-regions [7]. Many city-oasis communities were actively created in the region at that period, differing with its own culture—Kaunchinskaya, Otrar-Karatauskaya, Dzhetyasarskaya [8]. The period of great migrations is transformed genetic and social structure of the Southern area. The newly arrived Turkic tribes fought for regional hegemony, as a result during the V-XII centuries in the region formed distinct medieval states: such as the Turkic Khaganate, followed by the Western Turkic Khaganate. Further north: the Turgesh Khaganate, the Karluk state, the Karakhanid state, the Kara-Kitai Khanate, and the Naiman Khanate; further south: the Karluk Khaganate and the Khorezmkhash Empire; and further west—the Oguz state [7]. Beginning of the VIII century, the Southern area falls under the influence of Islam. Then in the XIII century, the Southern region became part of Genghis Khan’s Empire and remained under his descendants’ rule until the nineteenth century, when their rule in the form of the Kazakh Khanate (1465–1847) was finally abolished as a result of the Russian Empire’s expansion to the Central Asia [9, 10]. The Kazakh Khanate was the successor state of the Golden Horde (Ulus of Jochi), which began extending its influence from Zhetisu to South Kazakhstan, then eventually encompassing the contemporary Kazakhstan regiom and adjacent republics [11].

The Southern region’s historical versatility was mirrored in the structure of its gene pool, a hallmark of which is the branching tribal organization in the Kazakh people. Kazakhs are united into hierarchically organized social groups (lineage, clan, tribe) by male-line ancestry-based genealogical kinship. A tight genetic link on the Y chromosome exists for the majority of members of such groupings [12, 13]. Therefore, the Y-chromosome is currently being successfully used in genetic genealogy researches [14].

The Y chromosome, which is transmitted solely through the male line, has a low effective pool size, very low genetic diversity, and significant interpopulation diversity, making it very sensitive to genetic drift and the founder effect [15]. These characteristics make it useful in human population and evolutionary genetics, as well as forensic science. Two forms of Y-chromosome variability are employed in these studies: STR loci (short tandem repeats) and SNP markers (single nucleotide polymorphism). The variability of STRs is manifested by a higher mutation rate than that of SNPs. This ratio serves as the molecular clock’s minute and hour hands [16]. The STR loci determine an individual’s haplotype. Males who are closely related have similar haplotypes, with the exception of homoplasia instances. SNPs identify Y-chromosome haplogroups. These are phylogenetic branches that connect clusters of related haplotypes. The Y-SNPs and Y-STRs analyses work in tandem to offer a full picture of paternal genetic affinities.

Polymorphism of the Y chromosome in the Kazakh population, particularly the southern area of Kazakhstan, is of interest both on a regional scale of Central Asia [17] and at the local tribal level [18, 19]. It was discovered from the ancient Central Asian area of Transoxiana that two-thirds of the gene pool of southern Kazakhs (examined sample *N* = 780) is haplogroup C2-M217, which is often found among the Konyrat (88%) and Alimul (75%) tribes. A strong founder effect is also evidenced in studies of 12 tribes of Kazakhs in the Southern area (*N* = 567 samples [18] and *N* = 460 samples [19]). There is an exception to the rule: several more ancestors from other Y-chromosome haplogroups were identified for the clans of the steppe clergy (kozha and sunak), which, according to traditional genealogy, descend from a fellow tribesman of the Prophet Muhammad [17]. At the same time, the J1-L859 variant belonging to the Quraysh tribe of the Prophet Muhammad was not detected. The steppe clergy’s genealogy was based not on biological kinship, but on spiritual heritage passed down from the teacher of Islam, missionaries from various populations, to his disciples [17]. In general, the findings of the haplogroup diversity study, taking tribal organization into consideration, show that the gene pool of Southern Kazakhstan was established by not only genetically related, but also relatively distant tribes [18]. This is also true for the whole Kazakh population, as demonstrated by a recent study that used molecular dispersion analysis, where the differences across tribes account for over 20% of genetic variance [20].

However, while the primary findings of prior research were addressed in terms of Kazakh tribal organization, they had little effect on the geographical characteristics of Y-chromosome diversity in the Southern region of Kazakhstan, which is no less important for performing forensic biogeographic searches [21]. The only report was that the Mantel test failed to detect a statistically significant correlation between genetic and geographic distances [20]. Although, it is worth taking into account the fact that all of these investigations were constrained by the weaker haplotype identifying strength based on the 17 Y-STR sites. Variability at 27 Y-STR loci is more informative, and a current database of Kazakhstani Y-chromosome variability is being established on its platform [22], which already encompasses Kazakhstan’s Western, Eastern, and Northern regions [23–25].

The purpose of this study is to increase Kazakhstan’s database of Y-chromosome variability by people from the country’s southern part and to analyze this variability in a geographical context (Almaty, Zhambyl, and Turkestan regions) utilizing 27 Y-STRs haplotypes and related haplogroups.

## Results and discussion

### Haplotype/allele frequencies and forensic parameters

27 Y-STR haplotype distribution in the Kazakh population of the Southern region of Kazakhstan (*N* = 468) are presented in Additional file 1. Haplotype frequency calculation found 427 distinct haplotypes (Additional file 2). Of these, 20 haplotypes are shared by two people, three haplotypes are shared by three people, two haplotypes are shared by four people, and the most frequent haplotype is shared by ten people. Forensic parameters were calculated that characterize the population of Kazakhs in the southern region of Kazakhstan: Discrimination Capacity (DC = 91.23%), Haplotype match probability (HPM = 0.0029) and haplotype diversity (HD = 0.9992) (Table 1). The indicators are comparable with the results for the general mixed samples of Kazakhs in Kazakhstan [22] and rank second in diversity after the population of Kazakhs from Karakalpakstan [24].

**Table 1:** Forensic parameters of 27 Y-STR haplotypes in the Kazakh populations

**Population**	Sample in Population	Number of distinct haplotypes	Frequency of unique haplotypes	Discrimination capacity	Haplotype match probability	Haplotype diversity
General Kazakh [22]	300	270	82%	90%	0.0042	0.9991
Northern Kazakh [23]	382	326	78%	85.34%	0.0044	0.9982
Western Kazakh Tribes [24]	405	366	84.44%	90.37%	0.0034	0.9991
Karakalpakstan Kazakh [24]	59	58	96.61%	98.30%	0.0175	0.9994
Eastern Kazakh [25]	246	207	75.61%	84.15%	0.0069	0.9971
Southern Kazakh (This study)	468	427	85.68%	91.23%	0.0029	0.9992

The genetic polymorphism of 27 Y-STR haplotypes was studied in the same samples of Kazakhs from Kazakhstan’s southern area, but within three regions: Almaty (*N* = 80), Zhambyl (*N* = 253), and Turkestan (*N* = 135). The Turkestan region indicated the most variability (HD = 0.9994), while the Almaty region revealed the least (HD = 0.9984) (Table 2).

**Table 2:** Forensic parameters of 27 Y-STR haplotypes in the Southern Kazakh populations

**Population**	Sample in Population	Number of distinct haplotypes	Frequency of unique haplotypes	Discrimination capacity	Haplotype match probability	Haplotype diversity
Almaty Region	80	76	91.25%	95%	0.0140	0.9984
Zhambyl Region	253	231	84.98%	91.30%	0.0048	0.9990
Turkestan Region	135	131	94.81%	97.04%	0.0079	0.9994

**Fig. 2:**
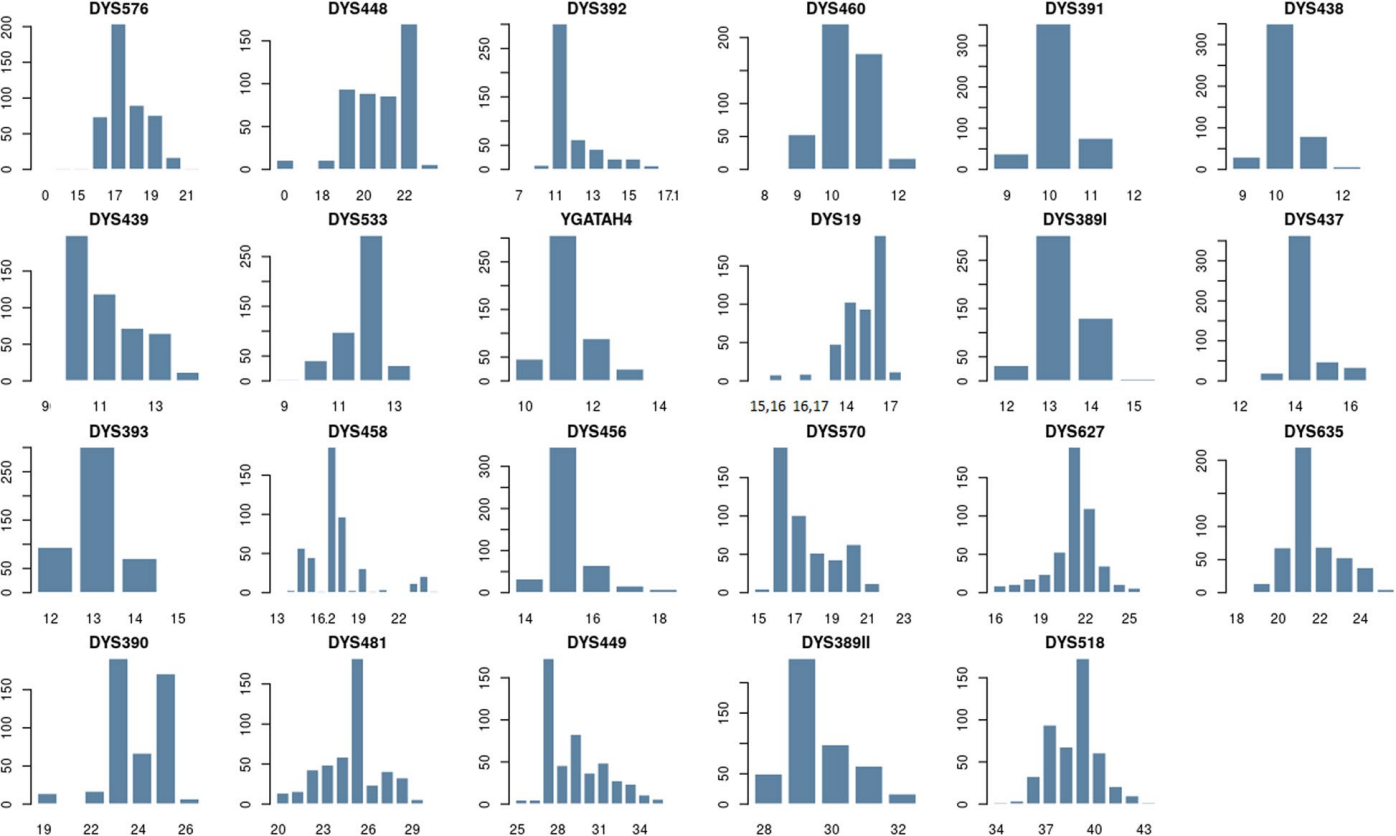
Distribution of allele frequencies for 23 single-locus Y-STRs in Kazakh population from Southern Kazakhstan. Horizontal scales – allelic values of the locus, vertical scale – allele occurrence

Distribution of allele frequencies and forensic parameters values for 23 single-locus Y-STRs in Kazakh population from Southern Kazakhstan (*N* = 468) presented in Additional file 3 and Fig. 2.

Y-STR profiling revealed 176 alleles at single-copy loci, the frequency of which varies from 0.002 to 0.778. The smallest number of allelic variants (*n* = 4) was found for the DYS389I, DYS391, DYS393 loci. For the DYS458 locus, the highest number of allelic variants was found (*n* = 16). Gene diversity (GD) in the samples of Kazakhs from the southern region of Kazakhstan varies from 0.378 for DYS437 to 0.80 for DYS449. On average, the gene diversity of rapidly mutating 8 single-copy loci (GD = 0.728) is higher than that of the standard 15 single-copy loci (GD = 0.571). The lowest indicators of Gene diversity in rapidly mutating loci are DYS460 (GD = 0.623) and DYS533 (GD = 0.533). Among the standard loci, DYS458 (GD = 0.769), DYS448 (GD = 0.758) and DYS19 (GD = 0.736) are characterized at the level of rapidly mutating loci.

Distribution of locus-specific haplotypes frequencies and forensic parameters values for DYS385a/b and DYF387S1 in Kazakh population from Southern Kazakhstan presented in Additional file 4. The DYS385a/b locus had 40 haplotype combinations of 12 distinct alleles while the DYF387S1a/b locus had 39 haplotype combinations of 11 different alleles. At the same time, the DYF387S1a/b locus had more gene diversity (GD = 0.917) than the DYS385a/b locus (GD = 0.879).

Abnormal alleles discovered in Kazakhs from Kazakhstan’s southern area are listed in Additional file 5. Microvariant alleles were discovered in 42 instances for the DYS458 gene and one case for the DYS392 locus. At the same time, microvariants for the DYS458 locus were indicative of the J1-M267 haplogroup in 98% of instances. There were 19 occurrences of duplication at the DYS19 gene, with 89% belonging to haplogroup C2a1a2-M48. There were 11 deletions at the DYS448 gene, with 91% belonging to the haplogroup C2a1a1b1-F1756. At the DYS576 locus, one deletion remains.

### Haplogroup frequencies

**Fig. 3:**
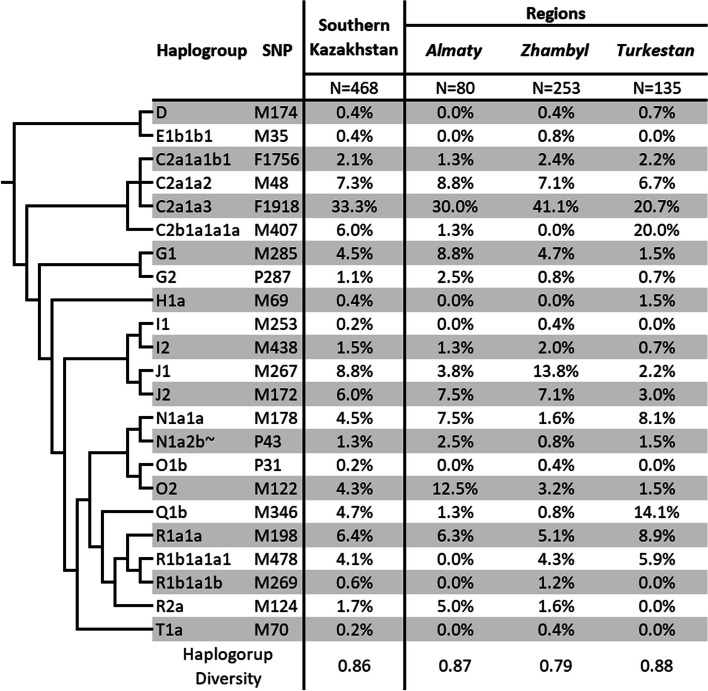
Frequencies of Y-chromosomal haplogroups in Kazakh populations from Southern Kazakhstan

The distribution of Y-chromosome haplogroups of the Kazakhs of South Kazakhstan is shown in Fig. 3. There is a high diversity of haplogroups (HD = 0.86). However, the majority of Y-chromosome diversity (81.2%) is spread across six haplogroups with a frequency of occurrence more than 5%: C2—48.7%; J1, 8.8%; R1a1a, 6.4%; J2, 6%; N1a, 5.8%; and G—5.6%.

C2-M217 is the most common haplogroup (48.7%), which is also shared by 50% of the Kazakh people [20]. Haplogroup C2-M217 is predominantly distributed in Northern and Eastern Eurasia, subdividing into two main branches C2a-L1373 [26] and C2b-F1067 [27], respectively. Both branches are present among the Kazakhs of South Kazakhstan. The C2a-L1373 branch is represented by 42.7% of the population. The C2b-F1067 branch is rare (6%), specifically the C2b1a1a1a-M407 sub-branch. It occurs in the study sample mainly in the Turkestan region (20%). The Konyrat clan has established itself in this area [28]. They are characterized by a significant founder effect within the subbranch C2b1a1a1a-M407 (86%) [19].

Numerous C2a-L1373 carriers in Eurasia belong to three major branches: M48, F1756, and F1918. The C2a1a2-M48 branch is widespread from Kamchatka (subbranch B90 [29]) through the Far East and Siberia (subbranch F5484 [30]) to the south of the East European Plain (subbranch F6379 [31]).

In Kazakhs appears the line C2a1a2a2a-F5485, the sub-branch within F6379. Its sublineage C2a'-Y15552 predominates (more than 70%) among the Kazakh tribes Alimuly and Baiuly from Western Kazakhstan [32]. The C2a1a2-M48 branch was found 7.3% of the time among Kazakhs in South Kazakhstan (Fig. 3). This frequency increases from 18 to 62% in the Aral Sea area, from east (Zhanakorgan district) to west (Kazalinsky district) [17].

The C2a1a1b1-F1756 branch is prevalent among Altai language families. Within it, two substantial subbranches are distinguished: C2a'-F8497 and C2a'-F3889 [33]. The initial C2a'-F8497 sequence is characteristic of Altai mountain people. The second C2a'-F3889 is found on the Mongolian Plateau and nearby areas to the west. F1756 is found in just 2.1% of Kazakhs in South Kazakhstan (Fig. 3). This branch, however, includes the genus Tore from Kazakhstan [34] and the genus Tusi Lu from China [35], both of which claim to be descendants of Genghis Khan. The first descend from Genghis Khan’s first son, Jochi, and the second from Khulgen’s sixth son.

The C2a1a3-F1918 branch is the most common (33.3%) among South Kazakhstan Kazakhs (Fig. 3). Its subbranch C2a1a3a-F3796 is notable for the possibility that its carrier was Genghis Khan and his male ancestors on the paternal side, who left a vast genetic trace across Asia in the shape of a Star-Cluster [36]. This cluster was later discovered to have grown much earlier and to be related with the ancient Mongolian tribe Nirun [37]. C2a1a3a-F3796 is often found among the Kazakh tribes of Uysun (40%) and Zhalaiyr from South Kazakhstan [19], and its ancestors may be traced back to the Nirun tribe. The Kerey tribe also contains C2a1a3a-F3796 [38].

The fraternal haplogroups J1-M267 and J2-M172 jointly account for 14.8% of the frequency of occurrence among Kazakhs in South Kazakhstan (Fig. 3). J1-M267 is found throughout West Asia and North Africa’s southern areas [39]. J2-M172 is also common among the populations of the Near and Middle East, Southern Europe, and the North Caucasus [40]. Both haplogroups are uncommon in Kazakhs, with 2.7% having J1 and 3.5% having J2 [20]. Ysty, on the other hand, has a 74% founder effect inside J1 [19]. This genus is found in the Zhambyl area [28], where it is most common (J1—13.8%) in this research.

The haplogroup R1a1a-M198 ranks third in terms of frequency of occurrence (6.4%) (Fig. 3). The haplogroup’s phylogenetics are separated between European and Asian branches [29, 41]. The branches M558 and M458 belong to the European branch, while Z93 belongs to the Asian branch. There is no information on the frequency of occurrence of subclades in the Kazakh population. The ancestral haplogroup R1a1a-M198 was found with high frequency in the Shanyshkyly (24%) and Oshakty clans (20%) [19]. Both clans are found in the Turkestan area [28], which has the highest frequency of this haplogroup—8.9% in our research. In the same Turkestan area, the fraternal haplogroup R1b1a1a1-M478 shows a significant frequency—5.9% among Kazakhs. R1b-M343 was discovered mostly among the Kipshak and Naiman tribes [20]. The settlement area of these tribes extends into Turkestan.

Haplogroup N1a-F1206 accounts for 5.8% of the Y-chromosome variability among Kazakhs in South Kazakhstan. Haplogroup N1a is widespread throughout the world, from the Far East to Eastern Europe, and several of its subbranches have a rather distinct geographical distribution [42]. Within the haplogroup N1a, two significant branches are distinguished: N1a2-L666 and N1a1a-M178. Both branches are present among South Kazakhstan’s Kazakhs. The Kazakh genera Sirgeli (80%) and Zhalaiyr (20%) showed a founder impact on the N1a1a-M178 branch [19]. According to Zhabagin et al. [28], Sirgely settle in the Turkestan area and Zhalaiyr in the Almaty region, which are the two places where the N1a1a-M178 branch occurs most often (8.1% in Turkestan and 7.5% in Almaty). It is quite uncommon (1.6%) in the Zhambyl area. The two sub-branches F4205 and M2118 of the N1a1a-M178 branch, which have previously been identified in Kazakhs, are of interest [42]. The second branch, N1a2-L666, is further differentiated into the P43 subbranch and the extremely uncommon M128 subbranch, which was previously discovered among Kazakhs (8.1%) [43]. In this study, only the P43 sub-branch was found, with a rare exception of 1.3%. Inside the P43 sub-branch, the B525 lineage was previously discovered in Kazakhs.

Haplogroup G-M201 completes the haplogroup spectrum, accounting for a sizable proportion (5.6%) of the Y-chromosome diversity among Kazakhs in South Kazakhstan. Its branch G1-M285 is most common in the Almaty region (8.8%). This branch (67%) is also found in the Argyn tribe [44, 45]. The settlement of the Argyn tribe is mainly in Northern and Central Kazakhstan [28].

Despite having a lower frequency of occurrence (5%) in the entire sample of Kazakhs from South Kazakhstan, haplogroups Q1b-M346 and O2-M122 have a considerable proportion in Turkestan (14.1%) and Almaty (12.5%), respectively. Previously, Haplogroup O2-M122 was relatively frequent among the Naiman tribe (52.3%) [20]. The Naiman tribe’s habitation territory includes Central and Eastern Kazakhstan, including the Zhetisu region [28]. The prevalence of the haplogroup in the Almaty region reflects the region’s closeness to Zhetisu.

### Population comparison analysis

The position of Kazakh populations in Kazakhstan’s Southern area is defined by three genetic positions—surrounded by geographical Kazakh populations, Kazakh tribal groupings, and Asian surrounding populations.

In the genetic position of geographical populations of Kazakhstan, 16 regions of Kazakhstan are represented: Abai (*N* = 28), Akmola (*N* = 43), Aktobe (*N* = 25), Almaty (*N* = 139), Atyrau (*N* = 211), East Kazakhstan (*N* = 97), Jetisu (*N* = 140), Karaganda (*N* = 52), Kostanay (*N* = 56), Kyzylorda (*N* = 71), Mangystau (*N* = 19), North Kazakhstan (*N* = 126), Pavlodar (*N* = 187), Turkestan (*N* = 204), West Kazakhstan (*N* = 66), Zhambyl (*N* = 332). The total sample includes 1796 Kazakh samples studied for 27 Y-STRs, including in our previous studies [22–25]. The pairwise genetic distance (RST) between the populations was estimated and is shown in Additional file 6. For Almaty, the nearby regions of Zhambyl (d = 0.0108) and Zhetisu (d = 0.0357) showed the shortest distances. These are Almaty (d = 0.0108) and Northern Kazakhstan (d = 0.0349) for Zhambyl. These are Karaganda (d = 0.0367) and Kostanay (d = 0.0514) for Turkestan. This might be evidence of “meridian” nomadism among Kazakhs, moving from south to north and north to south [46].

**Fig. 4:**
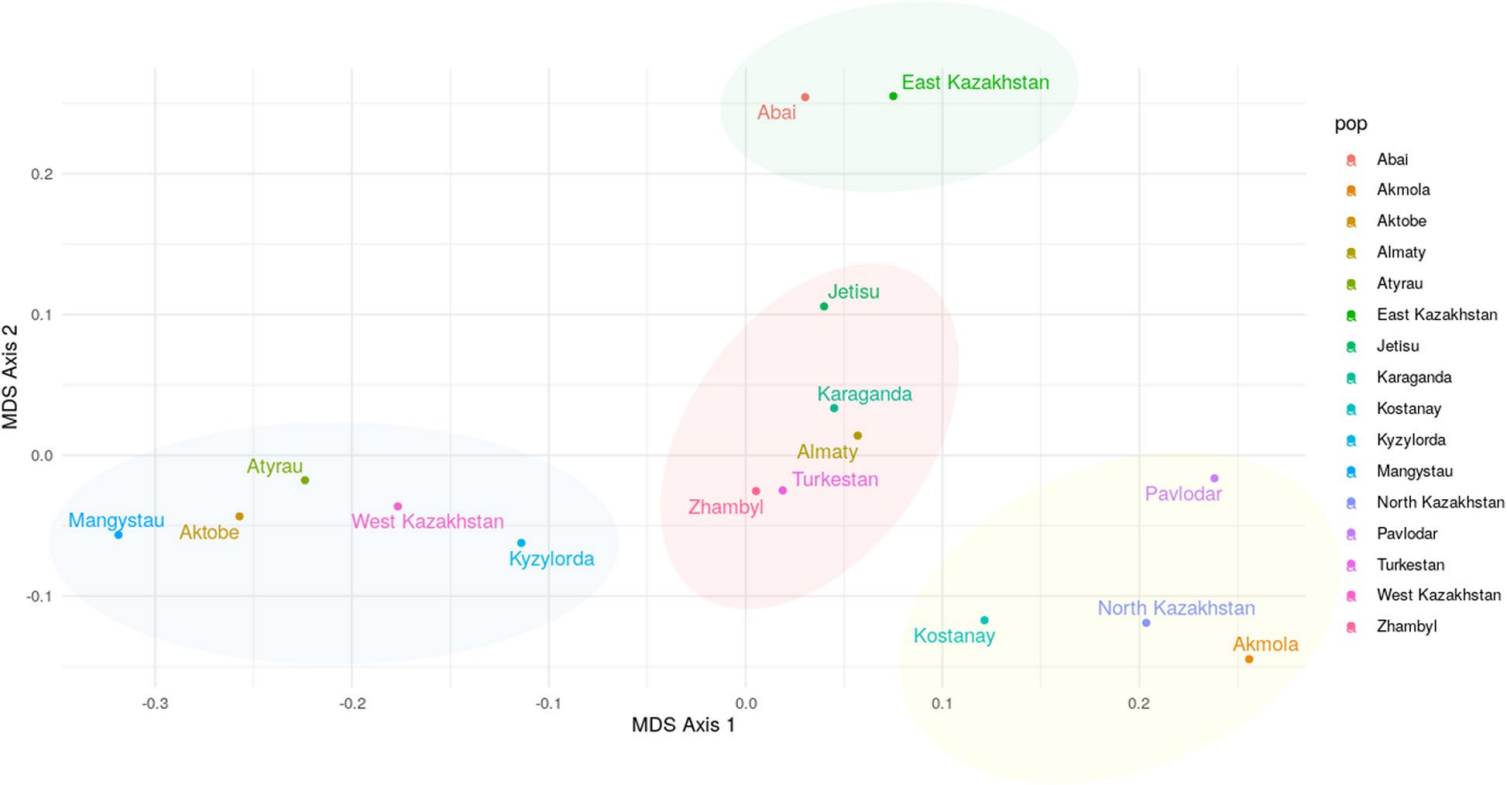
MDS plot based on Nei’s genetic distances between Kazakh populations on 23 Y-STRs

**Fig. 5:**
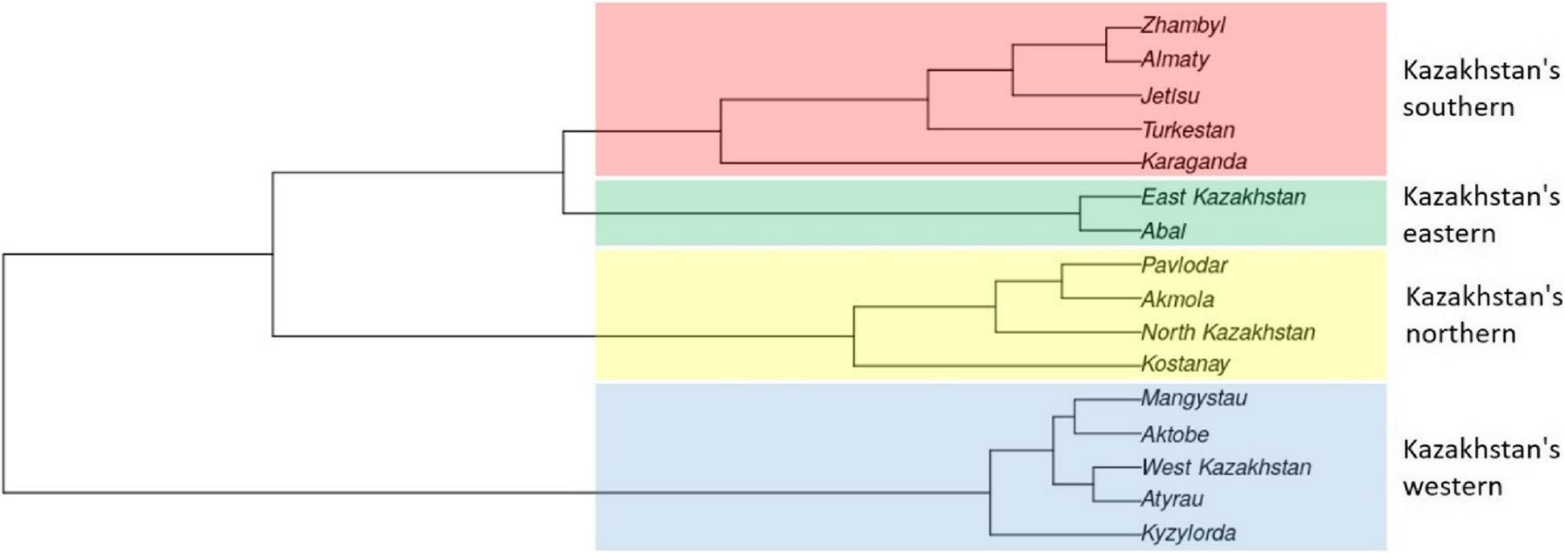
The dendrogram plot based on Nei’s genetic distances between Kazakh populations on 23 Y-STRs

The locations of the Kazakh populations in the genetic space were depicted using MDS (Fig. 4) and a dendrogram (Fig. 5) based on Nei’s genetic distances for 23 Y-STR loci. Populations are classified into four groups, which correspond to Kazakhstan’s southern (Zhambyl, Almaty, Zhetis, Turkestan, and Karaganda), northern (Palodar, Akmola, Northern Kazakhstan, and Kostanay), eastern (Eastern Kazakhstan, Abai), and western (Mangystau, Aktobe, Western Kazakhstan, Atyrau, and Kyzylorda) regions. Central Kazakhstan has yet to establish its own cluster.

**Fig. 6:**
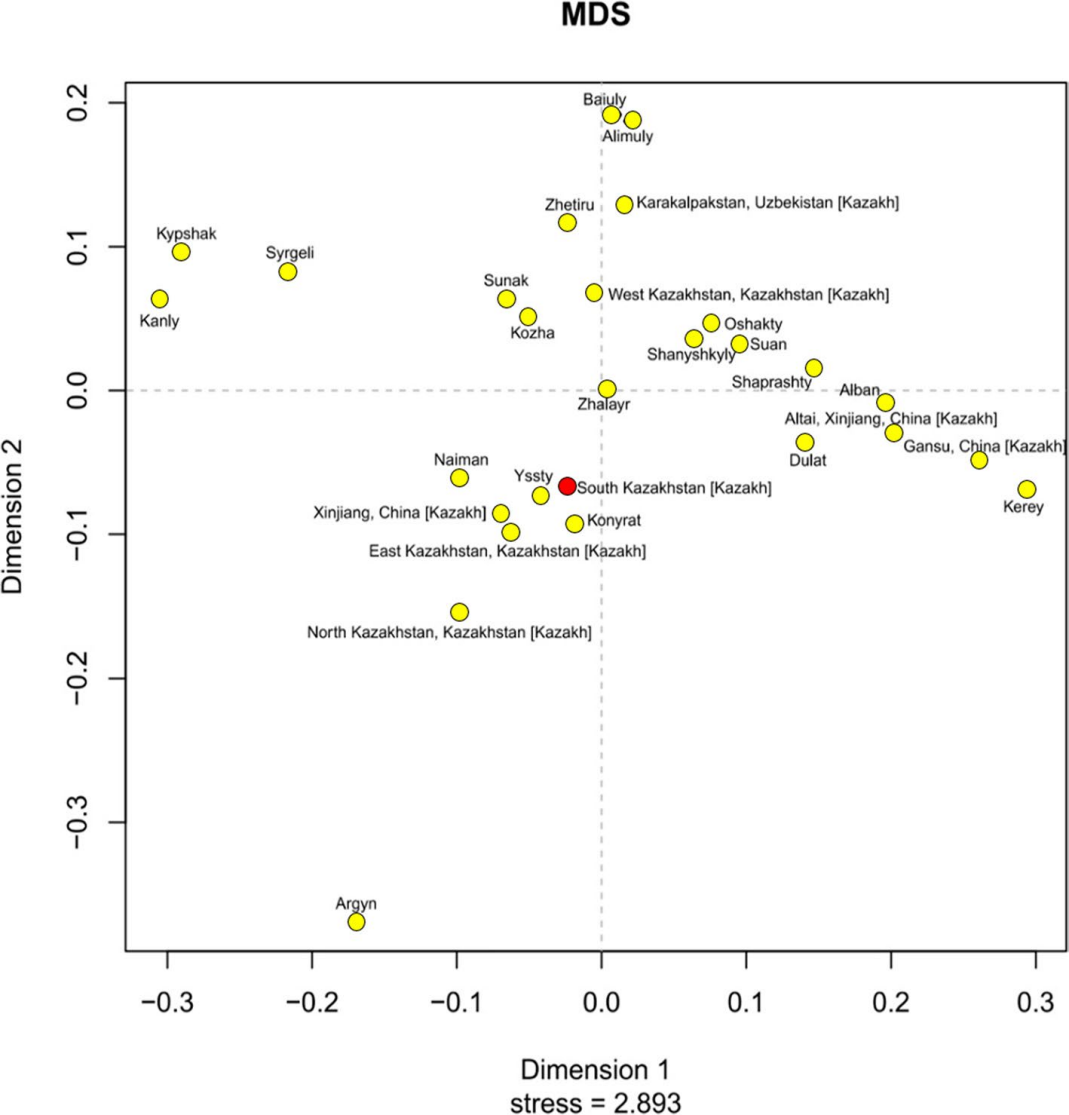
MDS plot based on pairwise genetic distance (RST) between Kazakh clans on 17 Y-STRs

In the genetic space of Kazakh tribal groups, 20 large tribes are represented: Alban (*N* = 68), Alimuly (*N* = 283), Argyn (*N* = 346), Baiuly (*N* = 572), Dulat (*N* = 261), Kanly (*N* = 70), Kerey (*N* = 154), Konyrat (*N* = 269), Kozha (*N* = 88), Kypshak (*N* = 37), Naiman (*N* = 162), Oshakty (*N* = 57), Shanyshkyly (*N* = 36), Shaprashty (*N* = 38), Suan (*N* = 49), Sunak (*N* = 35), Syrgeli (*N* = 48), Yssty (*N* = 72), Zhalayr (*N* = 210), Zhetiru (*N* = 181). Unfortunately, samples with known affiliations across 20 tribes are limited on the 17 Y-STR sites. The total sample includes 3036 Kazakh samples studied on 17 Y-STRs from previous studies [17, 19, 20, 22, 23, 32, 35, 38, 44]. The pairwise genetic distance (RST) between the populations was calculated and presented in Additional file 7. The population of the Kazakhs of the southern region of Kazakhstan is genetically closest to the Zhalaiyr (d = 0.0588), Shanyshkyly (d = 0.0711), Suan (d = 0.0836), Oshakty (d = 0.0851), Sunak (d = 0.0888), Dulat (d = 0.0965). The farthest genetic distance was determined from Argyns (d = 0.3446), Kypshaks (d = 0.2597), Baiulys (d = 0.2515) and Alimulys (d = 0.2256). The result is consistent with the fact that genetically close tribes are settled within the southern region of Kazakhstan. On the MDS plot, the populations of the Kazakhs of the southern region of Kazakhstan also clustered with the Kazakhs of China (Xinjiang) and the eastern Kazakhs of Kazakhstan, as well as with the tribes of Ysty, Konyrat, Naiman (Fig. 6). The area of settlement of these tribes also covers the considered Southern region of Kazakhstan.

**Fig. 7:**
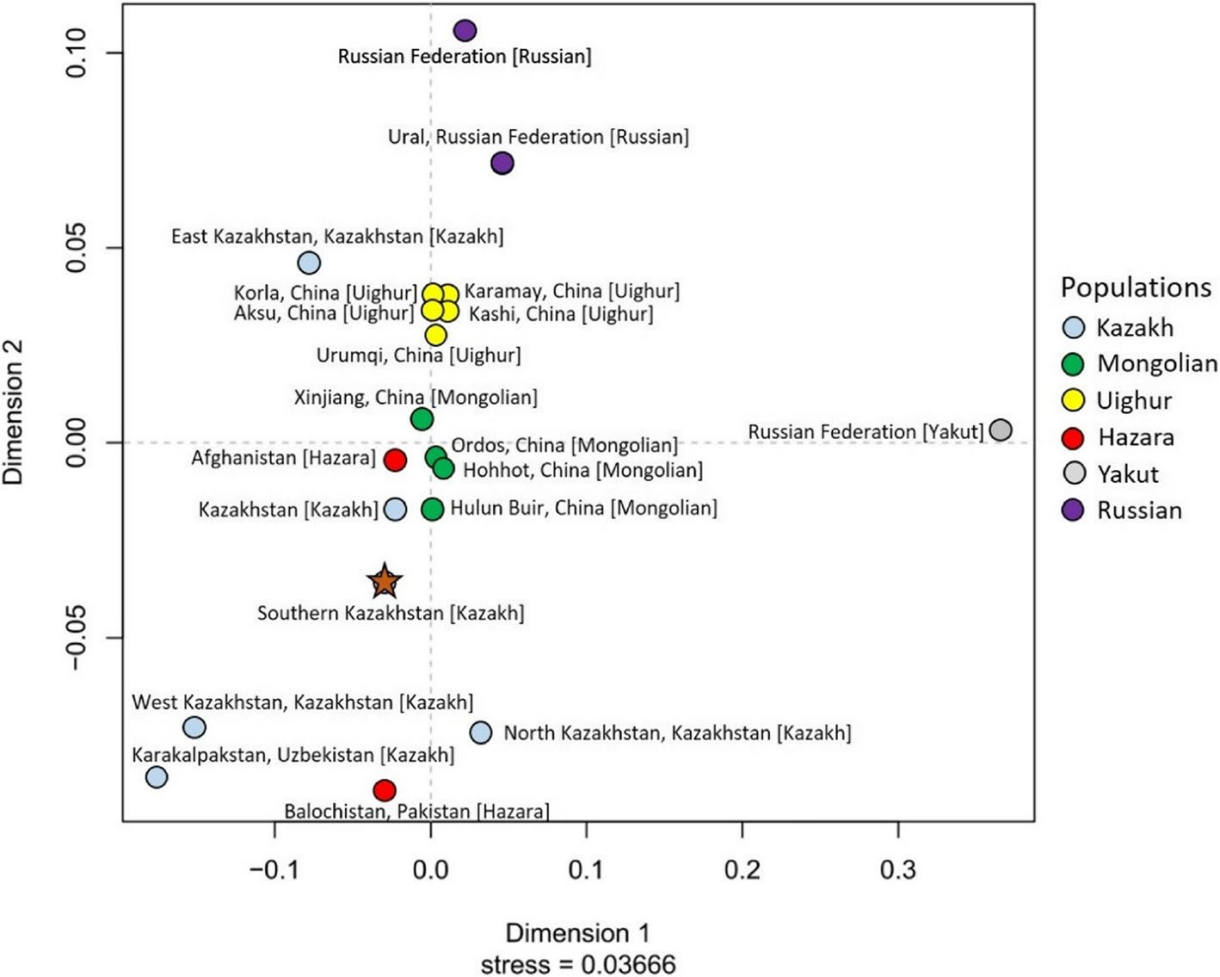
MDS plot based on pairwise genetic distance (RST) between Kazakh populations and neighboring populations used for comparison from YHRD on 27 Y-STRs

In the genetic space of Asia, populations of Kazakhs and culturally and historically close populations are represented, in total 19: Afghanistan [Hazara] (260 haplotypes); Hohhot, China [Mongolian] (240 haplotypes); Hulun Buir, China [Mongolian] (508 haplotypes); Ordos, China [Mongolian] (213 haplotypes); Xinjiang, China [Mongolian] (182 haplotypes); Aksu, China [Uighur] (150 haplotypes); Karamay, China [Uighur] (129 haplotypes); Kashi, China [Uighur] (77 haplotypes); Korla, China [Uighur] (141 haplotypes); Urumqi, China [Uighur] (49 haplotypes); East Kazakhstan, Kazakhstan [Kazakh] (246 haplotypes); Kazakhstan [Kazakh] (300 haplotypes); North Kazakhstan, Kazakhstan [Kazakh] (382 haplotypes); West Kazakhstan, Kazakhstan [Kazakh] (405 haplotypes); Balochistan, Pakistan [Hazara] (153 haplotypes); Russian Federation [Russian] (691 haplotypes); Ural, Russian Federation [Russian] (91 haplotypes); Russian Federation [Yakut] (34 haplotypes); Karakalpakstan, Uzbekistan [Kazakh] (59 haplotypes). The total sample includes 4310 samples studied from 27 Y-STRs and presented in YHRD (submission accession numbers for each population are given in Additional file 8). Pairwise genetic distance (RST) between the populations was calculated and presented in Additional file 8. There are no differences in terms of population between Kazakhs living in Kazakhstan’s southern area and those living in Kazakhstan generally. Kazakhstan [Kazakh] has the shortest genetic distance (d = 0.0139) with our samples. Further genetically close population are: Afghanistan [Hazara] (d = 0.0261), Hohhot, China [Mongolian] (d = 0.0591), Hulun Buir, China [Mongolian] (d = 0.0352), Ordos, China [Mongolian] (d = 0.0522), and Xinjiang, China [Mongolian] (d = 0.0402). The results are visualized in Fig. 7.

### Median-joining network for Southern Kazakhs

**Fig. 8:**
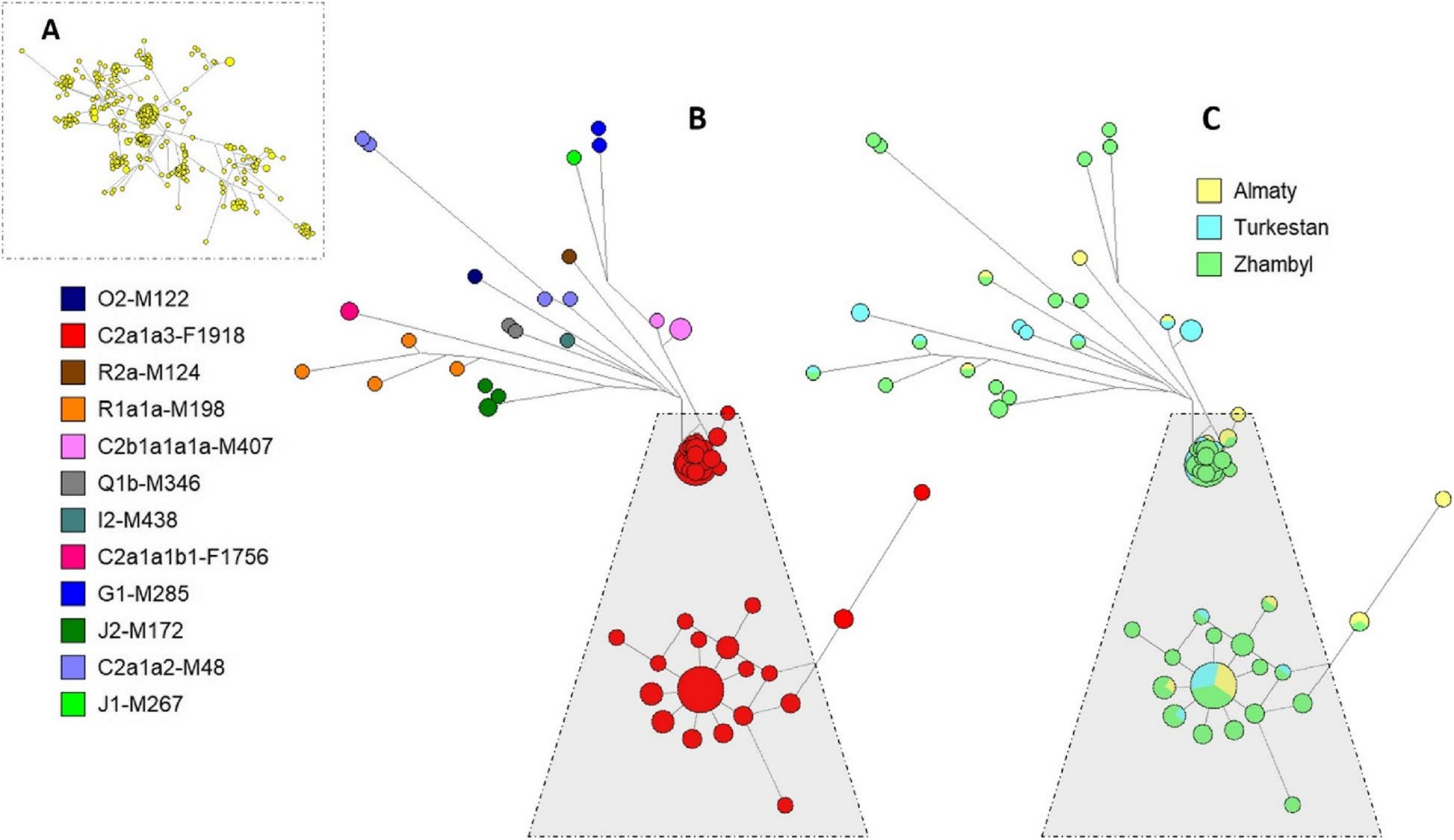
Median-joining network for the haplotypes of 468 Kazakh from Southern Kazakhstan (**A**), constructed from data on 23 Y-STRs. The colours representing the haplogroups (**B**) and geographical region (**C**). Circles represent haplotypes (Frequency > 1 criterion active for **B** and **C**), with the area proportional to sample size, and lines between them proportional to the number of mutational steps

**Fig. 9:**
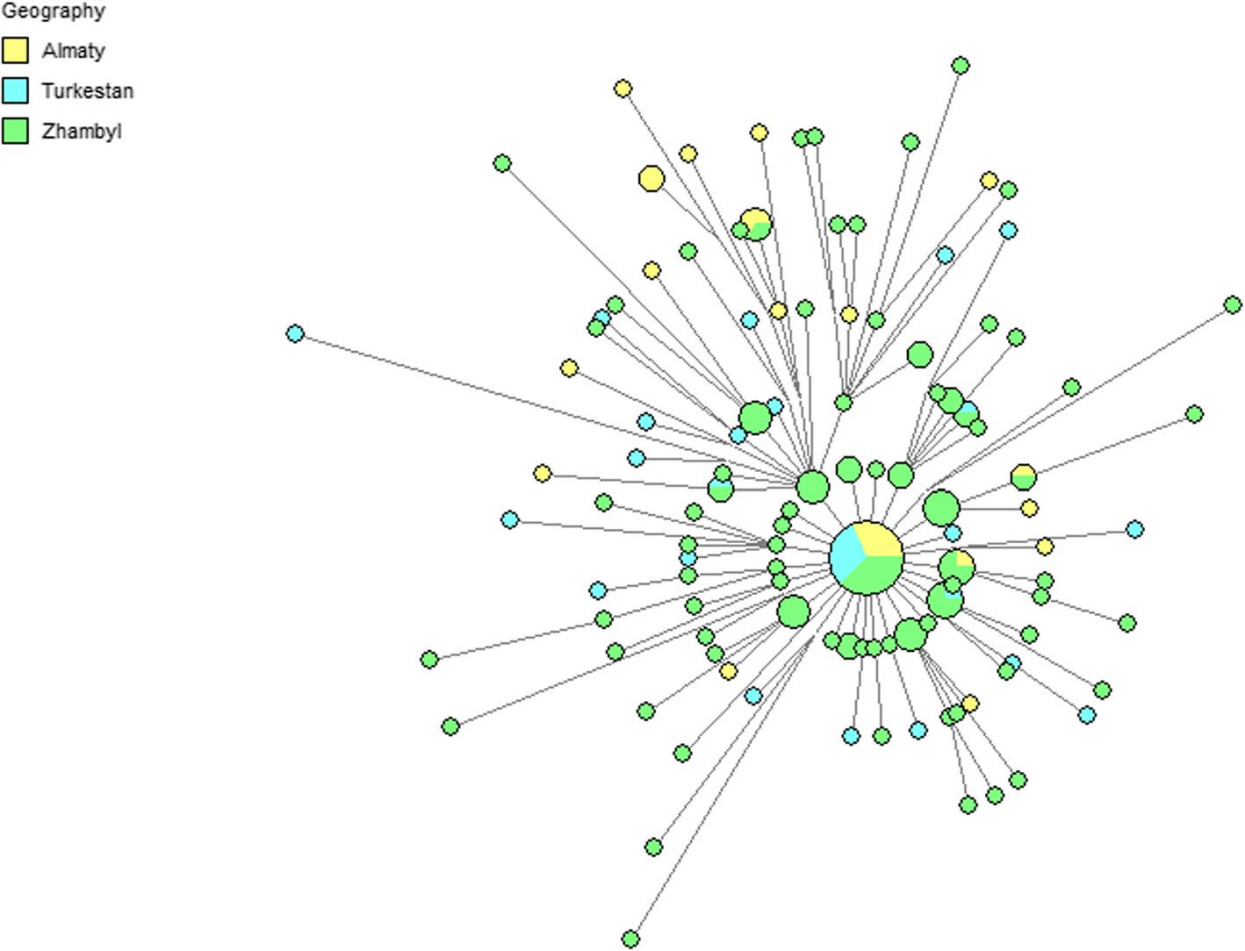
Reduced-median network of Kazakh from Southern Kazakhstan – C2a1a3 based on 23 Y-STRs. Circles represent haplotypes, with the area proportional to sample size, and lines between them proportional to the number of mutational steps

A median network was built to investigate the link between the 23 Y-STR haplotypes in our data set (Fig. 8A). Multiloci (DYS385a/b, DYF387S1a/b) were not included. A simplified version of the median network excludes single haplotypes and indicates haplogroup (Fig. 8B) and geography (Fig. 8C) affiliations. In total, 12 haplogroup clusters were discovered on Fig. 8B. On Fig. 8B and C, the cluster C2a1a3-F1918 is additionally presented in an enlarged format for clarity. With the exception of C2a1a1b1-F1756, C2b1a1a1a-M407, Q1b-M346 and R2a-M124, the haplotypes of the Zhambyl area are cluster-forming everywhere, according to the Fig. 8B and C. While in Almaty haplotypes cluster-formers are R2a-M124, and for the Turkesten haplotypes are C2a1a1b1-F1756, C2b1a1a1a-M407, Q1b-M346. In this scenario, haplotypes from three regions are equally represented as the founding haplotype of the cluster of haplogroup C2a1a3-F1918. This is the largest cluster among the Kazakhs of Southern Kazakhstan. This suggests, that expansion of the haplogroup C2a1a3-F1918 to the three regions of the Southern Kazakhstan happened at the same time period. Figure 9 visualizes the strong founder effect of this cluster, which includes 156 haplotypes and shows that derived haplotypes from the founder haplotype are mainly represented by individuals from the Zhambyl region. Large haplotype diversity in the Zhambyl region according to haplogroup C2a1a3-F1918 clearly reflects the presence of a preserved tradition of patrilocal and patrilineal families in this region.

## Conclusions

This study added 468 new male haplotypes from the Almaty, Zhambyl, and Turkestan areas of South Kazakhstan to the database of diversity of 27 Y-STR loci in the Kazakh population. As a result, we now have a database containing 1796 male Kazakh samples from 16 areas of Kazakhstan. This significantly improves the possibilities for applying data in human population genetics research and forensic-medical analyses. The study’s findings allowed for a regional analysis of the variation in Kazakhs’ Y chromosomes, revealing four primary groups in the south, north, east, and west. The Kazakhs of South Kazakhstan had genetic affinities with the Hazaras of Afghanistan and the Mongols of China. The population of southern Kazakhs clusters with tribes from the same region in the genetic space of tribal groupings. This is also supported by the represented data on Y-chromosome haplogroup variability. Phylogenetic analysis reveals a high diversity of haplotypes in the Zhambyl region. The identified genetic polymorphism and indicators of forensic parameters in the future, taking into account more distinct geographical units and tribal groupings, may allow for high biogeographic resolution for Y-chromosome markers in the Kazakh population.

## Methods

### Sample collection

This study was permitted by the Ethics Committee of the Asfendiyarov Kazakh National Medical University for the M. Aitkhozhin Institute of Molecular Biology and Biochemistry (No. 6 of 29 October 2012) in accordance with the principles of the Helsinki Declaration (1964). Unrelated male Kazakhs with ancestors who had resided in South Kazakhstan for at least three generations were asked to participate in the study as volunteers. In three regions of southern Kazakhstan, blood samples were taken from 468 Kazakh men. Each participant provided their informed consent by signing a consent form in order to participate in the study.

### DNA extraction and quantification

DNA was extracted from saliva samples of participants using the Wizard(R) Genomic DNA Purification Kit (Promega, USA). Fluorimetry was used to determine DNA concentration using a Quantus Fluorometer (Promega, USA) and a QuantiFluor(R) ONE dsDNA System (Promega, USA) kit. The quality of the DNA was determined using a NanoDrop One Spectrophotometer (ThermoFisher Scientific, USA).

### Y-STR fragment analysis with capillary electrophoresis

Fragment analysis of 27 Y-STR loci (DYS576, DYS389I, DYS635, DYS389II, DYS627, DYS460, DYS458, DYS19, YGATAH4, DYS448, DYS391, DYS456, DYS390, DYS438, DYS392, D YS518, DYS570, DYS437, DYS385, DYS449, DYS393, DYS439, DYS481, DYF387S1, DYS533) was performed using the Yfler Plus Amplification Kit (ThermoFisher Scientific, USA) on a SimpliAmp Thermal Cycler (ThermoFisher Scientific, USA). PCR products were separated by electrophoresis using LIZ600 size standard v2 (ThermoFisher Scientific, USA) in a Hi-Di Formamide Master Mix (ThermoFisher Scientific, USA) on an 8 capillary Applied Biosystems 3500 genetic analyzer (ThermoFisher Scientific, USA). ThermoFisher Scientific’s GeneMapper IDx v.1.6 software was used to examine the electropherograms. Samples with non-standard patterns, off-ladder and microvariant alleles were repeated. Haplotypes were used to determine haplogroups with the Nevgen Y-DNA haplogroup prediction tool [47]. Subsequently, genotyping was performed according to 23 Y-SNPs candidate for haplogroups (M174, M35, F1756, M48, F1918, M407, M285, P287, M69, M253, M438, M267, M172, M178, P43, P31, M122, M346, M198, M478, M269, M124, M70) on a QuantStudio5 instrument (ThermoFisher Scientific, USA) using TaqMan assays (ThermoFisher Scientific, USA).

### Data analysis

Haplotype frequency was calculated using Arlequin program ver 3.5 [48]. Number of distinct haplotypes, frequency of unique haplotypes, discrimination capacity, haplotype match probability and haplotype diversity were calculated by direct counting. The haplotype diversities were computed as HD = n*(1 − ∑p_*i*_^*2*^*)/(n − 1)*, where n is the sample size and p_i_ is the frequency of *i-*th haplotypes [49]. The sum of squared observed haplotype frequencies was used to determine the haplotype match probability (HMP). The ratio between the total distinct haplotypes and the number of haplotypes was used to calculate discrimination capacity (DC).

Forensic parameters such us Random match probability (RM), Power of Discrimination (PD), Gene diversity (GD), Polymorphism Information Content (PIC), Power of Exclusion (PE), Typical Paternity Index (TPI) and frequency for each locus were calculated using the online STRAF 2.1.5 program [50]. The same software illustrated Nei’s genetic distances [51] using a dendogram and multidimensional scaling (MDS). The YHRD website’s “AMOVA and MDS” online program [52] was used to compute pair-wise genetic distances (RST) and multidimensional scaling (MDS).

Median-joining networks were constructed by the software NETWORK v5.0.1.0 and NETWORK Publisher v2.1.2.5 (Fluxus Technology Ltd., England) [53], using maximum parsimony option for post-processing [54]. Intermediate alleles with repeat numbers were rounded off to the nearest integer. he duplicated loci DYS385a/b and DYF387S1a/b were excluded from the network construction as it is not possible to associate particular alleles to specific copies.

Following the requirements for population genetic data [55], the haplotype and haplogroup data in the present study (*N* = 184) have been submitted to the YHRD (accession number YA006016, YA006018, YA006019). Control DNA 007 (ThermoFisher Scientific, USA) was used as a positive control and ddH2O was used as a negative control for each batch of genotyping. The laboratories passed the YHRD Quality Control Test (YC000343) to contribute to the haplotype data.

### Supplementary Information

**Additional file 1: Table S1.** The haplogroup and haplotype distributions of 27 Y-chromosomal STRs in Kazakh population from Southern Kazakhstan (*N*=468).

**Additional file 2: Table S2.** The haplotype frequencies of 27 Y-chromosomal STRs in Kazakh population from Southern Kazakhstan (*N*=468).

**Additional file 3: Table S3.** Allele frequencies and Forensic parameters values for 23 single-locus Y-STRs in Kazakh population from Southern Kazakhstan (*N*=468).

**Additional file 4: Table S4.** Locus-specific haplotypes frequencies and Forensic parameters values for DYS385a/b and DYF387S1 in Kazakh population from Southern Kazakhstan (*N*=468).

**Additional file 5: Table S5.** Allelic micro-variants detected in the Sothern Kazakhstan population.

**Additional file 6: Table S6.** Pairwise genetic distance (RST) between Kazakh population from Kazakhstan Regions on 27 Y-STRs.

**Additional file 7: Table S7.** Pairwise genetic distance (RST) between Geographical Kazakh populations and Kazakh clans on 17 Y-STRs.

**Additional file 8: Table S8.** Pairwise genetic distance (RST) between Kazakh populations and neighboring populations used for comparison from YHRD on 27 Y-STRs.

## Data Availability

Haplotype data has been uploaded to the YHRD (https://yhrd.org/details/contribution/6016, https://yhrd.org/details/contribution/6018, https://yhrd.org/details/contribution/6019). Supplementary data associated with this article can also be found in the supplementary materials.

## Abbreviations

GD: Gene diversity
HD: Haplotype diversity, Haplogroup diversity
MDS: Multidimensional scaling
PCA: Principal component analysis
PCR: Polymerase chain reaction
PD: Power of discrimination
PIC: Polymorphism information content
PM: Match probability
Rst: Estimation of population differentiation assuming a stepwise mutation model
SNP: Single nucleotide polymorphism
STR: Short tandem repeat
YHRD: Y-chromosome haplotype research database
